# Exerkines and myokines in aging sarcopenia

**DOI:** 10.3389/fendo.2025.1592491

**Published:** 2025-07-29

**Authors:** Huan Wang, Wenbi He, Peishan Chen, Haozhe Wang, Huiguo Wang, Lin Zhu, Xiaoguang Liu

**Affiliations:** ^1^ Sport and Health, Guangzhou Sport University, Guangzhou, China; ^2^ Research Center for Innovative Development of Sports and Healthcare Integration, Guangzhou Sport University, Guangzhou, China

**Keywords:** sarcopenia, exerkines, myokines, exercise, skeletal muscle

## Abstract

Aging sarcopenia is an unavoidable condition that affects the majority of older adults in their later years. Exercise has been extensively researched as an effective intervention for sarcopenia. In particular, the release of exerkines and myokines during physical activity has beneficial effects on the body, which, as mediators, offer a novel therapeutic strategy for elucidating how exercise enhances skeletal muscle mass and function. In this review article, we summarize how exerkines exert protective effects on aging skeletal muscle mainly through the following mechanisms: (1) mediating energy diversion to skeletal muscle, ensuring more energy supply to the muscle; (2) enhancing the activity of skeletal muscle satellite cells to promote muscle repair and regeneration; (3) upregulating the expression of genes associated with muscle regeneration and, at the same time, inhibiting the expression of those genes that contribute to the atrophy of skeletal muscle; and (4) improving the function of the neuromuscular junction to improve the neural control of skeletal muscle. These combined effects constitute the protective mechanism of myokines on aging skeletal muscle.

## 1 Introduction

The growing number and proportion of older individuals within the total population is one of the most significant demographic challenges worldwide. The United Nations projects that, by 2030, 16.9% of China’s population will be over the age of 65, while the global elderly population is anticipated to exceed 1.5 billion by 2050 (1, 2). The U.S. Census Bureau and the National Center for Health Statistics predict that by 2040, 80.8 million Americans will be 65 years old, representing approximately 21.6% of citizens. Among them, 14.4 million will be 85 years old, which will be a 123% increase from 2017 (3). With the improvement of people’s life expectancy, society has entered the stage of aging gradually, which results in various diseases in the elderly and leads to the aggravation of the social economy and medical burdens (4). Additionally, aging is a multifaceted biological process characterized by a progressive decline in physiological function and an increased susceptibility to disease, and this process is accompanied by both functional and structural changes within the organism (1), such as increased genetic instability, loss of protein homeostasis, and cellular senescence-induced neurodegenerative changes during aging (5). However, decreases in skeletal muscle mass and strength are common in the older population. This progressive decline, known as sarcopenia, leads to impaired physical mobility and disability in older adults (6). The pathogenesis of aging sarcopenia is accompanied by a reduction in skeletal muscle mass and impaired contractile function and it also involves systemic metabolic, inflammatory, and endocrine abnormalities (7). When the rate of protein decomposition in skeletal muscle exceeds its rate of synthesis, this imbalance leads to muscle atrophy; thus, maintaining protein homeostasis during aging is essential to prevent muscle loss (8, 9). However, with aging, a combination of disturbances in muscle homeostasis and neuronal degeneration results in the preferential loss of type II (fast) muscle fibers. This selective loss is accompanied by a reduction in motor units, which further exacerbates the weakening of muscle strength, ultimately resulting in muscle weakness and bradykinesia (10, 11).

Exercise, as a non-pharmacological intervention, has great potential to improve age-related diseases (12). In particular, resistance exercise is effective in activating the nervous system and accelerating muscle protein synthesis to increase skeletal muscle mass and strength (13). During exercise, skeletal muscles secrete various molecules that participate in the crosstalk between organs and play an active role in neurological, metabolic, cardiovascular, and immune processes (12, 14). These small molecules synthesized and secreted by skeletal muscle are known as myokines (15). Furthermore, during and/or after exercise, peptides, metabolites, and nucleic acids released into circulation are exerkines (16), which regulate numerous physiological and pathological processes within the body, ultimately influencing metabolism to promote health (17). Some skeletal muscles secrete factors that are both exerkines and myokines, such as interleukin-6 (IL-6), irisin, fibroblast growth factor 21 (FGF21), and brain-derived neurotrophic factor (BDNF) (17). Mobility exerkines are key molecules mediating the link between exercise, metabolism, and inflammation, and minor changes induced by exercise may affect the whole body (17).

Exerkines have ameliorative effects on age-related diseases and may be a potential avenue for improving them through exercise. Of these, myokines are most closely related to skeletal muscle (18, 19). Therefore, the aim of this paper is to review the research progress on exerkines and aging sarcopenia in recent years, to explore the causal relationship between sarcopenia and myokines’ plasma levels, and to provide a reference for in-depth research on the homeostasis of skeletal muscle and rejuvenation therapy of skeletal muscle in the elderly.

## 2 Overview of aging sarcopenia

Sarcopenia was first recognized as an age-related loss of lean body mass, and in 2010, it was recognized as a separate condition (20). The European Working Group on Sarcopenia in Older People (EWGSOP) defines sarcopenia as a progressive, generalized skeletal muscle disease involving reduced muscle mass and dysfunction, the prevalence of which increases with age (21, 22). According to its pathogenesis, it can be categorized as primary sarcopenia, which is a loss of muscle mass and dysfunction associated with aging, or secondary sarcopenia, which has significant predisposing factors, such as chronic diseases and malnutrition (23, 24). Sarcopenia evolves from muscle atrophy to muscle dysfunction and then to muscle strength decline (21). In addition, we should also emphasize the difference between sarcopenia and skeletal muscle atrophy. Muscle atrophy occurs when muscle mass and fiber size decrease (25). At this point, it is important to emphasize that sarcopenia is a progressive decline in muscle mass, strength, and function with age due to environmental or genetic factors (26). However, both conditions involve reductions in skeletal muscle mass and muscle fiber size, and both are associated with an imbalance between protein synthesis and degradation within the muscle fibers (25). The mechanisms of skeletal muscle atrophy involve the ubiquitin–proteasome and autophagy-lysosome machinery, IGF1-protein kinase B-forkhead box O (IGF1–AKT–FoxO) signaling, inflammatory cytokines, nuclear factor-kappa B (NF-kB) signaling, and other signaling pathways. The underlying mechanisms of sarcopenia have been extensively studied, and we can confirm that reduced physical activity, hormonal imbalances, decreased absorption, and chronic inflammation affect the release of myokines and influence the development of sarcopenia (27).

**Figure 1:**
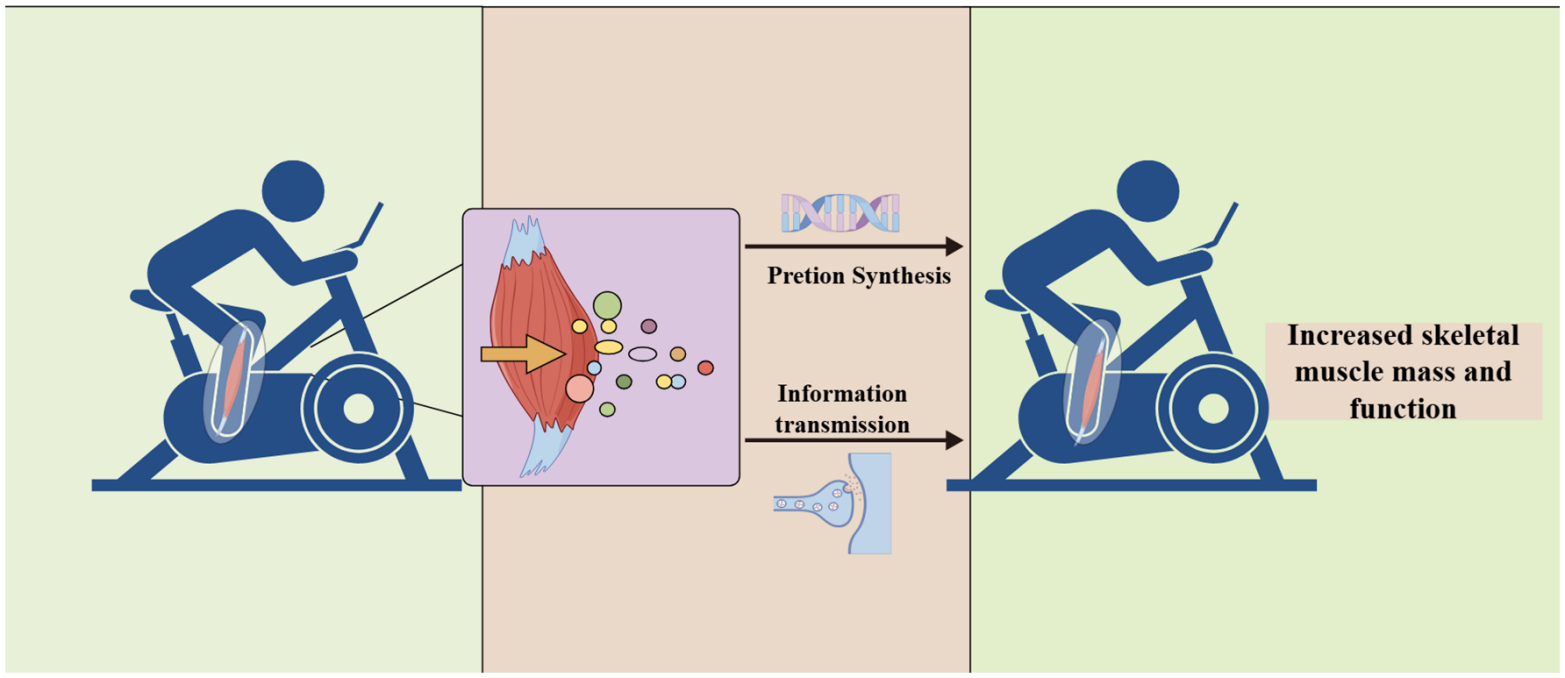
Mechanisms of aging sarcopenia.

Additionally, the pathogenesis of aging sarcopenia may involve satellite cell senescence, motor neuron loss, neuromuscular junction inactivity, mitochondrial function, hormonal status, and abnormal muscle factor production (28) (**Figure 1**). First, with aging, protein synthesis and catabolism are abnormal. When skeletal muscle protein catabolism exceeds synthesis, a negative protein balance occurs with muscle atrophy (8). Second, the breakdown of muscle homeostasis and neuronal degeneration lead to satellite cell senescence and a reduction in the number and size of skeletal muscles (11, 29, 30). Finally, mitochondria play a crucial role in muscle mass and function, and their dysfunction is a driver of sarcopenia (29, 31–33). Changes in the above factors also affect patient mobility. However, physical activity and exercise are effective countermeasures against skeletal muscle aging and delay or prevent metabolic muscle damage (34). Because exercise stimulates and promotes skeletal muscle contraction, it releases a variety of myokines that maintain skeletal muscle mass and enhance skeletal muscle regeneration (18, 35). Furthermore, changing myokine signaling in patients with sarcopenia leads to muscle atrophy and decreased fitness, while muscle atrophy also decreases myokine expression (36, 37).

## 3 Overview of exerkines

**Figure 2:**
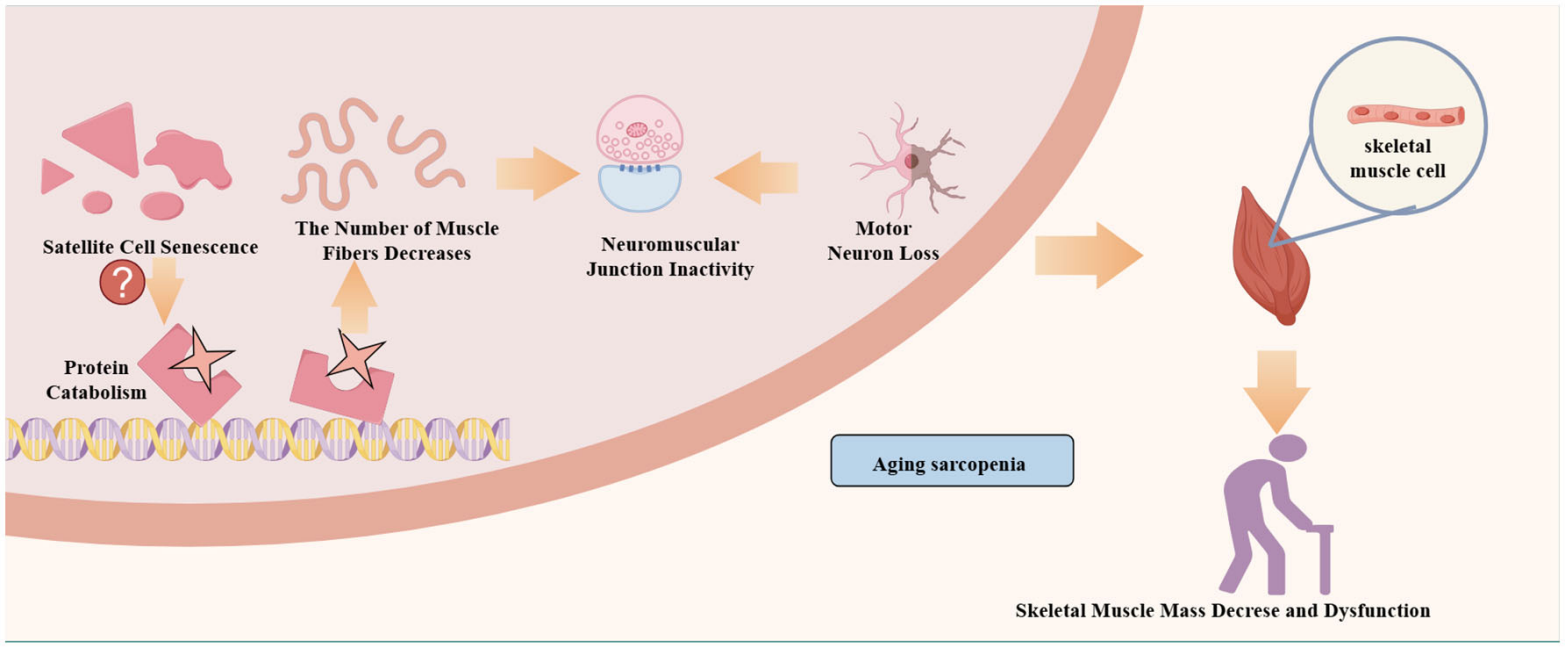
Classification of exerkines.

During or after exercise, tissues secrete different kinds of peptides, lipids, and nucleic acid substances, which are exerkines (16). Based on the site of exerkine release, researchers have designated exerkines released from skeletal muscle as myokines, those from the heart as cardiokines, those from the liver as hepatokines, those from white adipose tissue as adipokines, those from brown adipose tissue as batokines, and those from neurons as neurokines (16) (**Figure 2**). Exerkines can be secreted directly into the circulation or indirectly with the help of extracellular vehicles. Meanwhile, their molecular targets and receptors are found throughout the whole body, including the heart, brain, pancreas, bones, fat tissue, immune system, and skeletal muscle (38).

Human and animal experiments have found that exerkines circulate in the blood to achieve the crosstalk between organs and tissues. In the cardiovascular system, exerkines enhance the metabolic health in the heart (39). The majority of cardiokines are also considered to be important mediators in maintaining cardiac homeostasis and responding to myocardial injury (40). Exercise promotes the secretion of cathepsin B from skeletal muscle, which, together with BDNF, mediates neuronal maturation and improves cognitive function in the brain (41). The beneficial effects of exerkines on the brain may manifest as improvements in mitochondrial function, reductions in oxidative damage, maintenance of protein homeostasis, and promotion of synaptic plasticity (42). Exerkines are involved in the function of osteoblasts and osteoclasts and improve metabolic disorders. For example, irisin and BAIBA promote bone anabolism, while myostatin (MSTN) affects the activity of osteoblasts and osteoclasts, and promotes bone catabolism (43, 44).

Skeletal myogenesis is a multistep process involving the proliferation, migration, and differentiation of myoblasts (45). Exerkines are involved in the process of skeletal myogenesis, and even a single exerkine acting on multiple organs promotes skeletal myogenesis synergistically (46). For example, irisin affects adipose tissue and pancreatic function and coordinates the energy supply to skeletal muscle indirectly (17). Various exerkines act synergistically on a single organ to maintain homeostasis in the internal environment of skeletal muscle. Presently, we are unable to elucidate the specific mechanisms and effects of these actions, which limits our ability to maximize the efficacy of exerkine interventions.

Exerkines are intricately associated with skeletal muscle function, and the identification or isolation of exerkines may offer a novel strategy for specific diseases. Because of the diversity of exerkines, we classified them according to their release sites (see **Table 1**). Additionally, we conducted a comprehensive search of databases such as PubMed, Web of Science, and Sci-Hub to provide an extensive overview of the molecular relationship between exercise and skeletal muscle. For the myokines that have been studied extensively and have great potential for treating aging sarcopenia, we will provide a detailed explanation of their role in sarcopenia in the following section.

**Table 1:** Kinds of exerkines related to skeletal muscle.

Types	Names	Primary Role	References
Myokines	SPARC	SPARC is upregulated during skeletal muscle growth, regeneration, and pathological conditions. In SPARC knockout mice, although the recovery of injured skeletal muscle strength following fatigue was diminished, reparative processes persisted.	(223)
Myokines	Myonectin	Myonectin mitigates skeletal muscle dysfunction via an AMPK/PGC 1α-dependent mechanism. In aging sarcopenia, the degradation of myonectin results in heightened mitochondrial dysfunction within denervated skeletal muscle, exacerbating muscle atrophy.	(63)
	Musclin	Musclin enhances skeletal muscle endurance by promoting mitochondrial biogenesis.	(224)
	SDF-1	Simultaneous pretreatment of skeletal muscle with SDF-1 and IL-4 resulted in improved morphology and larger, more evenly distributed muscle fibers upon recovery of skeletal muscle after its injury. IL-4 and SDF-1 significantly enhanced the regenerative function of skeletal muscle by modulating the function of adipose tissue-derived stromal cells (ADSCs).	(225)
	FGF19	FGF19 ameliorates obesity-induced skeletal muscle atrophy, regulates skeletal muscle mass, and mitigates skeletal muscle wasting potentially.	(226)
	Sestrin	The sestrins (sestrin 1 and sestrin 2) are integrators of anabolic and catabolic metabolic pathways, which protect skeletal muscle from aging-related atrophy. Skeletal muscle proteolysis was increased when sestrins were specifically knocked down in mouse skeletal muscle. In contrast, when skeletal muscle overexpressed sestrin 1 or 2, the aging-induced loss of muscle mass and strength was partially reversed.	(227, 228)
	TNF-α	TNF-α acts on myocytes directly to enhance catabolic processes and promote skeletal muscle protein degradation. Additionally, TNF-α impairs the regenerative response following muscle injury through its effects on satellite cells. The exogenous administration of TNF-α stimulates both myocyte proliferation and satellite cell activation. Conversely, knockdown of TNF-α facilitates C2C12 myoblast proliferation.	(229)
	IL-8	IL-8 serves as an enhanced skeletal muscle anti-catabolic metabolic factor that reduces atrogin and MuRF1 expression and increases myotube length and diameter. The addition of IL-8 to primary myoblast culture dishes stimulated the expression of skeletal muscle hypertrophy-associated protein (myocilin).	(230)
	IL-7	IL-7 mRNA and protein levels are elevated during the differentiation of satellite cells into myotubes. IL-7 may stimulate satellite cell migration, indirectly leading to reduced differentiation and affecting skeletal myogenesis.	(231)
Cardiokines	Metrnl	Metrnl protects cardiac function and is protective against aging or inflammatory skeletal muscle diseases.	(232)
	CTRP9	CTRP9 overexpression ameliorates cardiomyocyte apoptosis and fibrosis while attenuating adverse cardiac remodeling.	(233)
	MG53	MG53 is involved in the protective effects against myocardial ischemia/reperfusion injury, which also regulates insulin sensitivity and energy metabolism in skeletal muscle.	(234, 235)
	Apelin	Apelin is an important regulator of cardiac and skeletal muscle homeostasis, and its absence leads to premature cardiac failure and sarcopenia.	(160)
	Follistatin-like 1	Promoting endothelial cell function and blood vessel growth in skeletal muscle and improving cardiac function after myocardial infarction.	(236, 237)
	Angiopoietin 1	Angiopoietin 1 promotes angiogenesis, enhances the survival of skeletal muscle satellite cells, and facilitates post-injury skeletal muscle regeneration.	(238)
	Fractalkine	Fractalkine improves skeletal muscle regeneration.	(239)
	Myonectin	Myonectin improves age-related skeletal muscle dysfunction (see the myokines section for specific functions).	(63)
	HGF	HGF may be involved in ischemic skeletal muscle regeneration by regulating muscle innervation and bioenergetics.	(240)
	VEGF	VEGF promotes capillary growth in skeletal muscle.	(241)
Adipokines	Adiponectin	Adiponectin improves glucose utilization and fatty acid oxidation in C2C12 myocytes and increases glucose uptake in skeletal muscle.	(242)
	Leptin	Leptin regulates adipogenesis in bone marrow mesenchymal stromal cells and specifically modulates skeletal muscle mass and contractile function. In conditions of fat mass deficiency, leptin secretion mitigates the decline in skeletal muscle mass and strength.	(78, 243)
	RBP4	RBP4 correlates with the presence and severity of aging sarcopenia. Knockdown of RBP4 attenuated denervation-induced fat infiltration and skeletal muscle atrophy while decreasing expression of the atrophy markers Atrogin-1 and MuRF1 and increasing expression of the myogenesis regulators MyoD and myoglobin. Lowering RBP4 levels may represent a promising therapeutic strategy for the prevention and treatment of muscle atrophy.	(244)
	Sfrp5	Sfrp5 attenuates insulin action in adipocytes under normal conditions and mitigates the inflammatory response in TNF-α-treated adipocytes, but not in skeletal muscle cells. The effects of Sfrp5 regarding inflammation and insulin resistance may depend on the specific site of action and metabolic context.	(245)
	Catecholamines	Catecholamines suppress the activity of the ubiquitin–proteasome system (UPS) and atrophy-related genes, all of which are via cAMP-dependent functions in skeletal muscle.	(246)
Hepatokines	Lactate	Lactate inhibits mitochondrial fatty acid uptake in skeletal muscle via malonyl coenzyme A and CPT1 inhibition. It also controls energy substrate partitioning in skeletal muscle.	(247)
	Fetuin-A	Fetuin-A participates in the downregulation of lipocalin disruption of mitochondrial energetics in skeletal muscle.	(248)
	Angptl	Angptl family member deletion causes skeletal muscle fat accumulation and insulin resistance with reduced whole-body energy expenditure in mice.	(249, 250)
	Follistatins	Follistatin overexpression induces skeletal muscle hypertrophy, increases muscle weight and torque production, and attenuates age-related degeneration at the neuromuscular junction in mice.	(177)
	LECT2	LECT2 induces insulin resistance in skeletal muscle.	(251, 252)
	SeP	SeP is implicated in sedentary-induced skeletal muscle atrophy. Excessive SeP adversely affects insulin secretion from the pancreas and diminishes insulin sensitivity in skeletal muscle.	(253, 254)
	Chemerin	Chemerin reduced insulin-stimulated Akt1 phosphorylation and activation of 5’AMP-activated protein kinase (AMPK) in the skeletal muscle and induced insulin resistance in skeletal muscle. Overexpression of chemerin in mice decreased skeletal muscle mitochondrial content and increased mitochondrial autophagy. Chemerin treatment of C2C12 myotubes increased the production of mitochondrial reactive oxygen species.	(255)
Neurokines	Irisin	Irisin’s main role in the brain is to protect brain function. The effect of irisin on skeletal muscle is summarized in the following section.	(256)

Secreted protein acidic and rich in cysteine, SPARC; Stromal cell-derived factor-1, SDF-1; Fibroblast growth factor 19, FGF19; Tumor necrosis factor-α, TNF-α; Interleukin-7, IL-7; Interleukin-8, IL-8; C1q tumor necrosis factor–related protein 9, CTRP9; Mitsugumin 53, MG53; Hepatocyte growth factor, HGF; Vascular endothelial growth factor, VEGF; Retinol binding protein 4, RBP4; Secreted frizzled-related protein 5, Sfrp5; Angiopoietin-like protein, Angptl; Leukocyte cell-derived chemotaxin 2, LECT2; Selenoprotein P, SeP. Partial exerkines are secreted by multiple organs, and we adopt the classification of that by researchers.

## 4 Overview of myokines

**Figure 3:**
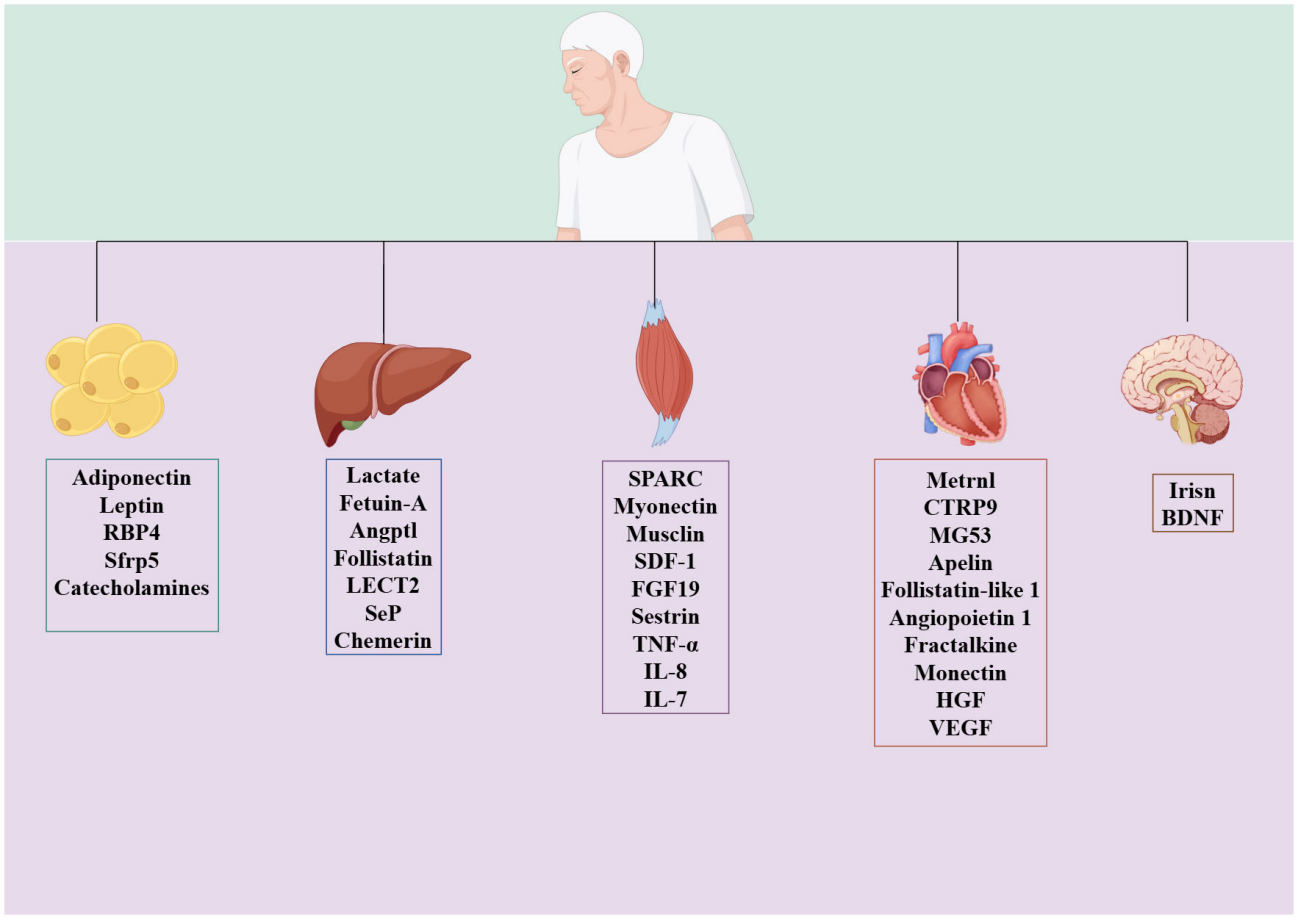
Production and action of myokines.

Skeletal muscle is one of the endocrine organs, and myocytes synthesize and secrete a variety of cytokines during contraction, named myokines, which exert autocrine and paracrine effects (15, 47). Multiple myokines have been identified by targeted analysis of skeletal muscle biopsy protein levels in a single acute exercise or long-term post-exercise population (18). Changes in the abundance of different myokines in the intermuscular fluid can be observed after exercise stimulation, and different exercise types stimulate different kinds of myokines (18). For example, muscles performing centripetal contractions induce the release of IL-6, interleukin-8 (IL-8), etc.; strength training induces the production of interleukin-15 (IL-15). A single exercise induces the release of IL-6, IL-1ra, and IL-8, and multiple strenuous exercises induce the release of tumor necrosis factor-α (TNF-α) (47, 48). In addition, different muscle fibers release different types of myokines; for example, glycolytic fibers mainly produce actin, angiopoietin, and Muscarinic Acetylcholine Receptors (mAChRs), whereas oxidative fibers mainly produce myosin and irisin (49–51). More than 600 myokines have been identified by non-quantitative tagging proteomic approaches. Some of them act in multiple organs throughout blood circulation to participate in metabolic processes, such as promoting glucose uptake, enhancing insulin sensitivity, improving cognitive functions in the brain, stimulating osteoblast differentiation, controlling blood pressure, and regulating myocardial contractility (52–54). Some are involved in combating acute inflammation caused by infection or low-grade inflammation due to aging (47). However, the most important physiological function of myokines is to protect skeletal muscle function and enhance skeletal muscle motility (18) (**Figure 3**).

During skeletal muscle cell proliferation, myoblasts secrete myokines that are more inclined to inhibit neurogenesis and adipogenesis, whereas during differentiation, myoblasts release some myokines that have the ability to promote myotube formation, vascular differentiation, and neurogenesis (35). Myokines, as signaling molecules, are important carriers in skeletal muscle and other organs, and have a complex regulatory network throughout the organism, including with the muscle (53), fat tissue (55, 56), the pancreas (57, 58), the brain (59), the vasculature (60), and bone (61), among others.

## 5 The role and mechanism of exerkines in aging sarcopenia

In this section, we aimed to elucidate the relationship between physical activity and aging sarcopenia from exerkines. When we searched Medline, PubMed, and Cochrane Library with the keywords “exerkines”, “aging sarcopenia”, and “skeletal muscle”, we found that a large number of exerkines were related to skeletal muscle (outlined in **Table 1**). We found that many researches focus on exerkiens related to skeletal muscle regeneration or functionn, with fewer studies report that exerkines improve aging sarcopenia. We selected myonectin, Metrnl, adiponectin (ApN), and leptin, which are more related to aging sarcopenia. Consequently, we will conduct an exploration of the relationship between these exerkines and aging sarcopenia, with the objective of identifying a novel therapeutic direction for aging sarcopenia from a systemic perspective.

### 5.1 Myonectin and aging sarcopenia

Myonectin, also known as C1q tumor necrosis factor-α-related protein isoform 15 (CTRP15), has the function of maintaining body homeostasis (62). Myonectin knockout reduces muscle strength in aging mice, which exacerbates muscle atrophy by downregulating the AMP-activated protein kinase/peroxisome proliferator-activated receptor-gamma coactivator (PGC)-1α (AMPK/PGC-1α) pathway (63). When exogenous myonectin supplementation is added, skeletal muscle atrophy is prevented by the AMPK α2/PGC-1α4/IGF-1-dependent pathway (63); however, in human trials, there were no limiting differences in serum myonectin levels among 142 older adults with or without sarcopenia (62). Myonectin plays an important role in maintaining homeostasis and preventing muscle atrophy *in vivo*, but its serum levels in the elderly are not significantly associated with sarcopenia.

### 5.2 Metrnl and aging sarcopenia

Metrnl, also known as meteorin-like hormone, cometin, subfatin, and IL-39, is expressed in a variety of tissues, including the liver, heart, stromal cells, macrophages, spleen, and the central nervous system (64). Although Metrnl is released by a variety of tissues, Lee et al. (65) found that myofiber-specific expression of Metrnl is not necessary for muscle regeneration, but when produced from macrophages, it can promote muscle regeneration. Macrophages change during aging, in which impairment of innate immune signaling leads to age-related muscle degeneration when skeletal muscle regenerates (66). Skeletal muscle injury decreased the expression of Metrnl and improved muscle regeneration in aged mice (66). However, in young mice, Metrnl is expressed in macrophages and induces the expression of regeneration genes (IL-10, IL-6, and IGF-1) to activate the proliferation of satellite cells when skeletal muscle is damaged (67). Thus, the decline in muscle regenerative function may be associated with changes in Metrnl concentration induced by macrophage changes during aging. Serum Metrnl also positively correlates with weight loss and the severity of cardiac insufficiency in elderly patients with chronic heart failure (CHF) (68). Intraperitoneal injection of recombinant Metrnl in mice can activate the activating cyclic AMP/protein kinase A/Sirtuin1 pathway and reduce ischemia/reperfusion injury-induced cardiomyocyte apoptosis via activation of AMP-activated protein kinase/p21 activated kinase 2 signaling for reduction of ischemia–reperfusion-induced apoptosis in cardiomyocytes (68). Recent experiments have identified that Metrnl promotes myosatellite cell proliferation to achieve the initial stage of skeletal muscle regeneration and also has a muscle regeneration-promoting function in aged, skeletal muscle-atrophic mice.

### 5.3 Adiponectin and aging sarcopenia

The majority of ApNs are secreted by white adipose tissue, skeletal muscle cells, cardiac muscle cells, liver parenchyma cells, and osteoblasts. ApN can be classified as full-length ApN (fApN) and globular ApN (gApN) according to its structure and function (69). During skeletal muscle injury, immune cells are recruited to the site of injury and release elastase, which cleaves fApN to gAPN. ApN is a protective factor against aging sarcopenia, and it is negatively correlated with skeletal muscle density, physical function, and bone density (70, 71). We found that ApN improves aging skeletal muscle in two aspects. On the one hand, gApN induces the expression of myogenic differentiation antigen (MyoD) to promote myoblast proliferation and differentiation and activates myoblastogenin and myoregulatory factor 4 to promote muscle differentiation and fusion into multinucleated myotubes (69). On the other hand, the ApN/adiponectin receptor 1-AMPK axis mediates exercise-induced satellite cell proliferation and improves locomotor activity in aging mice (69). At the same time, the improvements exhibit muscle-type specificity. Tail vein injection of the ApN receptor agonist (AdipoRon) three times a week for 6 weeks in 25-month-old mice significantly enhances the function and metabolism of fast-twitch fibers but does not affect slow-twitch fibers (72). All in all, ApN affects mitochondrial metabolism, muscle fiber regeneration, and skeletal muscle autophagy against skeletal muscle dysfunction in aged individuals.

### 5.4 Leptin and aging sarcopenia

Leptin is mainly derived from adipose tissue and correlates with total fat mass and plays a key role in energy balance regulation, appetite control, insulin sensitivity, and glucose metabolism (73, 74). Higher leptin levels are associated with a higher risk of sarcopenia in the elderly (75). In a cross-sectional study of 4,062 subjects (≥69 years old), serum leptin levels were found to be associated with the risk of obesity-related sarcopenia (76). In contrast, Kao et al. (77) found that higher serum leptin levels were correlated with a lower risk of sarcopenia. Such contradictory findings may be related to the body fat content of the subjects, and leptin receptor sensitivity is higher in obese individuals. Elevated levels of leptin significantly influence fatty acid oxidation and lipid metabolism in skeletal muscle, consequently leading to skeletal muscle dysfunction (76). In order to explore the relationship between leptin, Collins created fat-free (FF) mice by crossing ApN-Cre mice. The resulting FF mice constitutively have a complete absence of adipose tissue from birth. FF mice have low leptin levels and exhibit muscle mass defects driven by fast fiber atrophy (78). The effects and mechanisms of leptin on skeletal muscle homeostasis are limited.

## 6 The role and mechanism of myokines in aging sarcopenia

**Figure 4:**
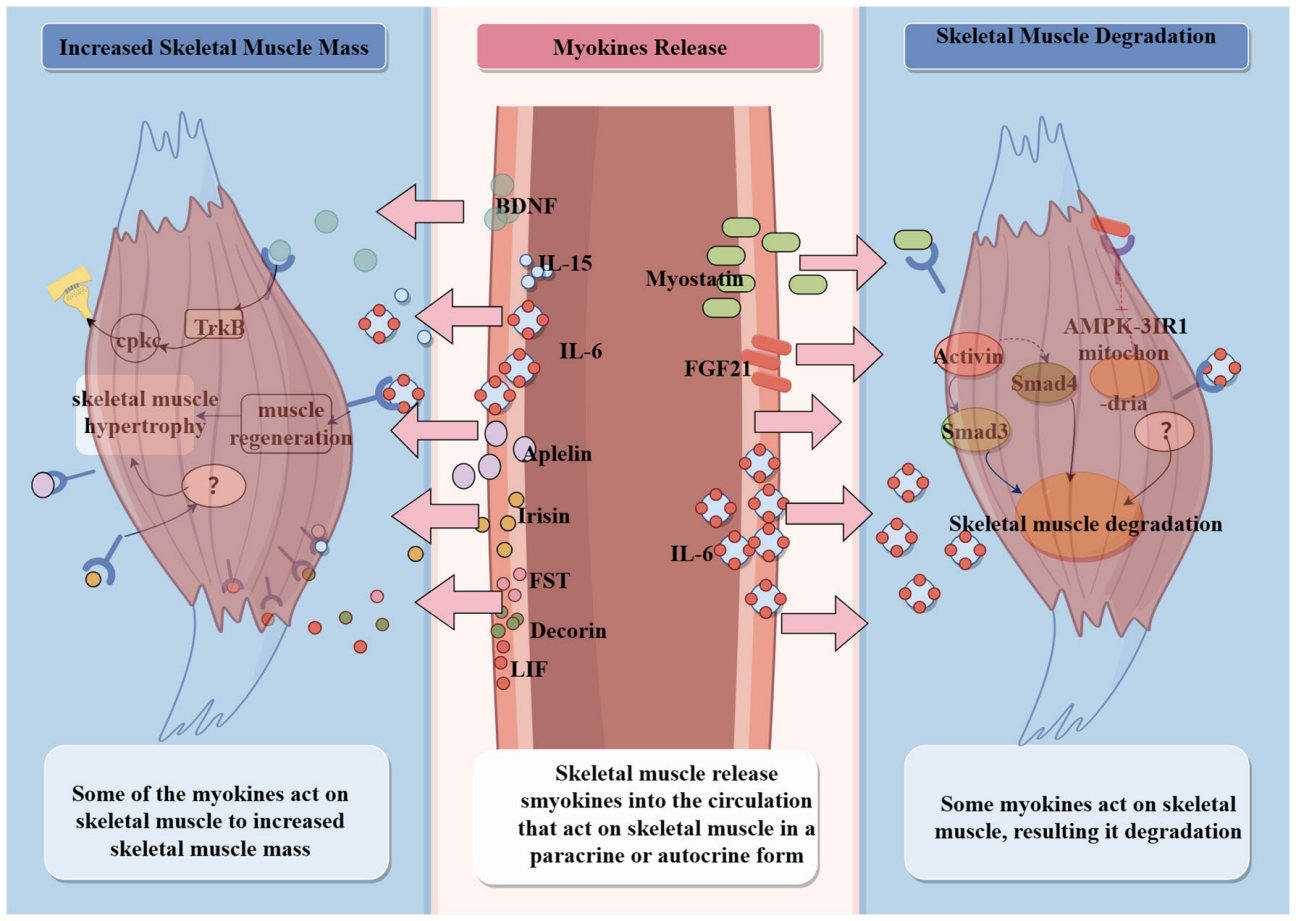
Mechanism of myokines action.

Exercise is an effective strategy to ameliorate sarcopenia. A number of studies have found that myokines improve skeletal muscle mass and strength. Based on the bidirectional effects of that on skeletal muscle mass, myokines can be categorized into two types that increase skeletal muscle mass and cause skeletal muscle atrophy. Myokines, as key mediators, may be a breakthrough in the study of the mechanisms by which exercise improves aging sarcopenia. The ameliorative effect of myokines on aging sarcopenia is characterized by the promotion of skeletal muscle protein synthesis, modulation of energy uptake in skeletal muscle, and enhancement of signaling at neuromuscular junctions (**Figure 4**).

### 6.1 Myostatin and aging sarcopenia

MSTN belongs to the transforming growth factor beta(TGF-β) superfamily (79). Skeletal muscle, cardiac muscle, adipose tissue, the brain, the kidneys, and even leukocytes can express MSTN (80–82). MSTN is first synthesized as an inactive precursor protein and hydrolyzed into active MSTN in two steps. First, the furin family removes potential MSTN complex signal peptides, and then bone morphogenetic protein 1 (BMP1)/Tolloid matrix metalloproteinases separate the muscle growth inhibitory ligand from the inhibitory N-terminal prepeptide structural domain, exposing the active site of MSTN binding to the receptor (83).

MSTN regulates skeletal muscle mass and function (84). Compared to younger men (mean age, 20 years), older men (mean age, 70 years) have approximately 100% higher expression of the MSTN gene, accompanied by an approximately 40% reduction in the cross-sectional area of type II muscle fibers. If the elders have elevated levels of serum MSTN, their grip strength will reduce by approximately 7.5% (85). It is hypothesized that MSTN level increases with age and loss of skeletal muscle, and a serum MSTN increase of 1 ng/mL is associated with an 11% increase in the odds of developing sarcopenia in older men (85–87). The mechanisms of MSTN that affect skeletal muscle mass are shown as MSTN binds to activins 2A and 2B and activates intracellular Smad2 and Smad3 signaling to negatively regulate skeletal muscle growth (88, 89). Specifically, the hydrolyzed active MSTN binds to the transmembrane hormone type IIB receptor (ActRIIB) through dimerization of disulfide bonds. This leads to the phosphorylation of Smad3 and Smad4, which then bind to activin receptor-like kinase 4 or 5 (ALK4 and ALK5) for signaling, affecting transcription factors such as the myocyte-specific enhancer factor (MEF2) and the myoblast determination protein 1 (MyoD1), which inhibit myoblast proliferation and differentiation (83, 90). Additionally, MSTN inhibits muscle hypertrophy through mammalian target of rapamycin (mTOR) signaling and increases muscle degradation through the forkhead box protein 01 (Fox01) pathway (83). As a negative regulator of muscle mass, MSTN knockdown or inhibition results in increased muscle mass. Therefore, blocking muscle growth inhibitor signaling or regulating its MSTN gene expression may be a therapeutic strategy for MSTN-related diseases, such as age-related sarcopenia.

### 6.2 Interleukin-6 and aging sarcopenia

IL-6 is a multifunctional cytokine released by multicellular cells during inflammation. IL-6 participates in innate and adaptive immune responses, and in the activation of anabolic and catabolic pathways to regulate cell growth, differentiation, and survival (91–93). IL-6 has three signaling pathways: the classical signaling pathway, the trans-signaling pathway, and the cluster signaling pathway (91, 94, 95). IL-6 regulates myosatellite cell proliferation and migration through these three signaling pathways and thus regulates physiological muscle hypertrophy (92). At the same time, IL-6 signaling is tightly regulated by feedback suppressors, such as suppressor of cytokine signaling, SH2-domain containing protein tyrosine phosphatase 2 (SHP2), and T-cell protein tyrosine phosphatase (TC-PTP), among others (92). Exercise induces IL-6 transcriptional upregulation through skeletal muscle contraction, contributing to increased serum IL-6 concentrations (91, 96).

Patients with aging sarcopenia have higher blood concentrations of IL-6 than healthy individuals, and elevated plasma IL-6 may increase fat deposition within skeletal muscle (97). In animal experiments, IL-6 injection into the tibialis anterior muscle of mice caused the upregulation of some genes related to immunity, and the downregulation of some genes related to energy metabolism (98). Pelosi and coworkers (99) constructed NSE/IL-6 transgenic mice and found that IL-6 overexpression in NSE/IL-6 mice resulted in a significant reduction in the rate of muscle growth in early postnatal life. In adulthood, these mice have severe muscle atrophy accompanied by low muscle mass and a significant reduction in muscle cross-sectional areas, individual muscle fiber cross-sectional areas, and total number of muscle fibers. Pelosi’s experiment demonstrated that elevated levels of IL-6 during the first and middle stages of life are an important factor in shifting muscle fiber type and skeletal muscle atrophy. However, elevated IL-6 has two opposite effects; slowly increasing the concentration of IL-6 in plasma result skeletal muscle atropht, but exercise-induced increase in plasma IL-6 promotes skeletal muscle regeneration (100, 101). On the one hand, the increased IL-6 concentration may activate satellite cells, enhancing muscle regeneration and growth (91, 102, 103). Additionally, IL-6 mediates short-term energy allocation, diverting energy to skeletal muscle (104). On the other hand, a slow and sustained increase in IL-6 inhibits skeletal myogenesis and protein synthesis (105), and accelerates skeletal muscle degeneration and atrophy (106–108). Whether the slow and sustained increase of IL-6 in the blood of elderly patients with sarcopenia causes damage to muscle fibers by influencing myofiber type transition and energy supply processes, thereby leading to atrophy of the whole skeletal muscle, still needs to be further explored.

### 6.3 Irisin and aging sarcopenia

In 2012, Bostrom et al. (109) found that exercise activation of PPAR-γ co-activator-1α (PGC-1α) upregulated its downstream target—fibronectin type III domain containing 5 (FNDC5). FNDC5 is a membrane protein present in the brain and skeletal muscle that is cleaved by an unknown protein hydrolase and releases irisin after exercise (110, 111). Animal studies have shown that irisin can enhance the activity of skeletal muscle satellite cells, reduce protein degradation, alleviate skeletal muscle fibrosis, and improve the stability of muscle membrane, thereby alleviating sarcopenia in different animal models, including those with denervated muscle loss, hindlimb suspension, and hereditary muscular dystrophy (46, 112, 113). During skeletal muscle differentiation, elevated levels of irisin upregulate p-Erk expression to promote the skeletal muscle protein synthesis pathway (46). During growth, intraperitoneal administration of irisin to 5-week-old mice resulted in an increase in skeletal muscle weight via the Akt and mTOR signaling pathways, subsequently translating this augmented muscle mass into enhanced grip strength. It may also downregulate the expression of Atrogin-1 and MuRF-1, thereby inhibiting the catabolic processes associated with skeletal muscle (46). During aging, Guo (12) found that irisin and its precursor FNCD5 in muscle tissue decreased with aging. Irisin knockout mice showed more severe muscle atrophy and smaller muscle mass and grip strength. However, injecting recombinant irisin protein into the abdominal cavity of aging mice can improve grip strength and muscle mass, thus improving symptoms of sarcopenia (12). Targeting the AMPK-PGC-1α-FNDC5 and the IGF-1/Akt/mTOR pathway with irisin prevents the onset of muscle disease in the elderly (114). In contrast, Baek et al. (115) found that there was no difference in plasma irisin levels between patients with aging sarcopenia and healthy older individuals, and even an increase in blood irisin levels could not change the state of reduced muscle mass, muscle dysfunction, and physical performance in patients with aging sarcopenia. These contradictory results may be related to the source of the subjects. The latter subjects came from outpatient geriatrics and endocrinology clinics. The baseline status of the subjects in the two studies was not consistent. In animal experiments, irisin produced positive effects on skeletal muscle mass and function in growth and developmental stages, and in aging. Replication of these results in animal studies in humans requires further investigation.

### 6.4 Fibroblast growth factor 21 and aging sarcopenia

FGF21 is a family of 22 related proteins that can be classified as six subfamilies based on genetic and functional similarities (116), which has a complex molecular mechanism of signaling, involving multiple FGF receptors (FGFRs) and a dedicated co-receptor, β-klotho (117–119). FGF21 expression is low in healthy skeletal muscle; however, fasting, endoplasmic reticulum stress, mitochondrial myopathy, and metabolic disorders induce increased expression of FGF21 in skeletal muscle, especially in dystrophic mice, where mRNA and protein expression of skeletal muscle FGF21 are significantly upregulated (120–122). In the elderly, elevated serum FGF21 levels were significantly associated with the risk of sarcopenia, low muscle mass, and low grip strength (123). Elevated levels of FGF21 are accompanied by decreased mitochondrial autophagy and skeletal muscle protein synthesis, resulting in loss of muscle mass and strength (124). Interestingly, inhibition of FGF21 levels in blood improves the aging phenotype (125). In animal experiments, skeletal muscle-specific FGF21 deficiency protects muscle from atrophy and weakness induced by starvation (121). These effects may be closely related to the state of mitochondria. FGF21 regulates mitochondrial function by activating the AMPK-SIRT1 pathway, which, in turn, activates peroxisome proliferator-activated receptor gamma coactivator-1α (PGC-1α), thereby affecting skeletal muscle energy supply (126, 127).

FGF21 secretion is increased in myasthenia gravis and mitochondrial stress states, and FGF21 regulates skeletal muscle glucose uptake (128) and protein synthesis (129). It has been hypothesized that increased FGF21 expression in sarcopenia may promote mitochondrial stress, reduce skeletal muscle glucose uptake, and disrupt skeletal muscle proteostasis. These states further stimulate FGF21 overexpression, thus creating a vicious cycle.

### 6.5 β-Aminoisobutyric acid and aging sarcopenia

Aminoisobutyric acid originates from different organs and can be classified as three isomers, α-aminobutyric acid (AABA), β-aminobutyric acid (BABA), and γ-aminobutyric acid (GABA). A pair of these isomers, which have the same molecular weight and related structures, mirror isomers, and are also known as enantiomers (130). β-Aminoisobutyric acid (BAIBA) is a PGC-1α-dependent myokine produced by skeletal muscle during exercise. It is mainly found as two enantiomers, L-BAIBA and D-BAIBA, which originate from different pathways (131–134). Skeletal muscle contraction stimulates young and old mice to release BAIBA to improve muscle mass and strength (132). In another study, plasma BAIBA levels were higher in adult subjects than in the elderly, and that BAIBA expression was regulated by PGC-1α (135, 136). It has also been suggested that BAIBA secretion is similar in young and old muscles, and the reduced BAIBA function may result from a significant decrease in its receptor with age (132). BAIBA has now been shown to promote osteoblast survival under oxidative stress and maintain bone quality (44, 132). The development of sarcopenia in older age groups is often accompanied by osteoporosis and dysfunction in muscle, leading to a reduction in skeletal muscle loading and bone mass (10). As sarcopenia is closely linked to endocrine and mechanical risk factors for osteoporosis, and muscles and bones act synergistically mechanically and biologically (10, 135), it is hypothesized that the effect of BAIBA on delaying skeletal muscle atrophy in sarcopenia in older adults may be through maintenance of bone mass and an increase in the muscle attachment points, attenuating the decline in muscle strength.

### 6.6 Brain-derived neurotrophic factor and aging sarcopenia

The mammalian neurotrophic factor family includes nerve growth factor (NGF), neurotrophin-3, neurotrophin-4/5, and BDNF (137). BDNF is secreted out of the cell as Pro-BDNF after being translated and cut into mature BDNF. BDNF and Pro-BDNF act on specific receptors in the nucleus and cell membrane (138, 139). Different skeletal muscles secrete BDNF in response to different exercise durations and intensities. Post-exercise plasma BDNF levels depend on exercise duration, its intensity, type of exercise, the level of previous training, and the functional status of the body (140–145). In skeletal muscle, BDNF regulates glycolytic fiber-type recognition, fatty acid oxidation, and satellite cell differentiation, and strengthens the neuromuscular junction (143, 146). BDNF takes part in the generation of regenerating muscle fibers after injury and is necessary for the formation of regenerating muscle fibers after injury or damage (147, 148). In contrast, plasma BDNF levels are significantly lower in patients with aging sarcopenia and in debilitated patients with diminished muscle strength and physical activity (149). In animal models, intramuscular injection of BDNF promotes functional repair after nerve injury. BDNF plays an important role in protecting the neuromuscular junction (150). The ameliorative effect of BDNF on aging sarcopenia involves muscle repair signal cascades and neuromuscular signaling connections. Activation of TrkB receptors by BDNF enhances presynaptic protein kinase C family (cPKCα, cPKCβI, and cPKCϵ), and activation of PKCs enhances synaptic vesicle fusion and neurotransmitter release (143), which enhances the functional innervation of the muscles (150).

Muscle wasting during aging is partly due to the retraction and death of motor neurons, resulting in the detachment of muscle fibers from neuronal innervation. Muscle fiber degeneration and muscle atrophy can only be avoided if these muscle fibers are re-innervated by neighboring neurons (151). Perhaps we may speculate that BDNF delays skeletal muscle atrophy during aging by enhancing neuromuscular connections in patients with aging sarcopenia.

### 6.7 Apelin and aging sarcopenia

Apelin belong to cardiac factor, myokine and adipokine (152), and is extensively distributed across various organs and tissues, including skeletal muscle, adipose tissue, the central nervous system, the gastrointestinal tract, lungs, liver, and heart (153). Apelin-13 and apelin-17 are the predominant isomers of apelin found in human plasma, with apelin-13 exhibiting greater biological activity and receiving more extensive research attention (154). Exercise and aging are important factors influencing apelin secretion. Exercise induces apelin, while overall levels decrease with aging (155, 156). However, the effect of exercise on apelin levels is controversial. One study demonstrated that prolonged aerobic exercise significantly elevated plasma apelin levels, whereas another study indicated that it did not influence apelin expression (152, 157).

Apelin enhances myocyte metabolism and stem cell function to stimulate skeletal muscle formation during aging (155, 158, 159). The Apelin/Apelin receptor system stimulates skeletal muscle stem cells through the Forkhead box 03–MuRF-1–Atrogin axis and simultaneously activates the AMP-activated protein kinase (AMPK) and P7050K pathways to promote protein production in myofibers, which together promote skeletal muscle regeneration (159). Knockdown of the apelin gene in the skeletal muscle of aged mice led to muscle mass reduction, muscle weakness, and motor dysfunction (160), whereas aged mice that are administered apelin or that are subjected to adenovirus-mediated enhancement of the apelin gene expression in skeletal muscle exhibit improved muscle function and hypertrophy of muscle fibers (155). Apelin-administered mice had increased expression of the markers Pax7, Myf5, and Myogenin in satellite cells and target muscle stem cells to promote muscle regeneration (156). Furthermore, apelin deficiency resulted in changes in skeletal muscle fiber types. Compared to wild-type mice, apelin knockout induces a shift from fast type II to slow-oxidizing type I fiber in mice and increases the proportion of MHC-1 type fibers (161). Additionally, apelin knockout mice exhibited a decreased number of mitochondria in myogenic fibers, a significant reduction in mitochondria-related enzyme activities, and diminished muscle tonic contractility and grip strength compared to wild-type mice (155).

Exercise-induced apelin enhances skeletal muscle function and alleviates sarcopenia, which makes apelin a potential target for the treatment of myofibrillar atrophy, muscle weakness, and oxidative stress in aging mice (159, 162). However, with age, both systemic and local apelin levels show a decreasing trend. Exogenous administration of apelin can lead to significant improvements in age-related pathologies. Apelin may serve not only as a novel tool for the early diagnosis of sarcopenia but also as a prognostic marker for evaluating the benefits of exercise in older adults.

### 6.8 Insulin-like growth factor-1 and aging sarcopenia

Insulin-like growth factor-1 (IGF-1) is an anabolic growth factor that facilitates tissue development, maturation, cellular adaptation, and regeneration during growth and development (163). In skeletal muscle, IGF-1 is secreted by muscle fibers into the extracellular matrix, subsequently binding to insulin-like growth factor binding proteins (IGFBPs) (164, 165). Age and exercise are key factors influencing circulating IGF-1 concentrations. Serum IGF-1 levels decrease with age, but exercise promotes ICF-1 secretion (166–169). Aerobic exercise, resistance exercise, whole-body vibration, and electrical stimulation all activate the IGF-1 pathway, increase protein synthesis and skeletal muscle mass, inhibit protein degradation and apoptosis, and enhance the exercise capacity of skeletal muscle in early aging mice (170). In animal experiments, IGF-1 knockout in mouse monocytes/macrophages resulted in impaired muscle regeneration after injury, with reduced size of regenerating muscle fibers, enlarged interstitial gaps, and deposition of lipid tissue (171). In contrast, IGF-1-overexpressing mice maintain high IGF-1 levels even in old age, thereby maintaining skeletal muscle function (6). IGF-1 achieves its protective effects on skeletal muscle by activating muscle signaling responses and skeletal myogenesis (6, 172). Elevated plasma levels of IGF-1 lead to IGF-1 Akt/Protein Kinase B-mTOR pathway stress, which promotes ribosomal biosynthesis and facilitates the formation of new myofibrillar proteins to provide a condition for skeletal muscle remodeling. Additionally, a high level of IGF-1 inhibits skeletal muscle via the ubiquitin ligases MuRF1 and MAFbx (173, 174). Skeletal muscle secretion of IGF-1 decreases during aging, which results in a decline in skeletal muscle mass and function. However, high plasma IGF-1 levels can increase muscle mass to decrease the incidence of aging sarcopenia.

### 6.9 Other myokines and aging sarcopenia

Follistatin (FST) is a multifunctional protein whose main function is to antagonize the TGB-β superfamily, such as the muscle growth inhibitors, activin, and BMPs (175). FST, as a cytokine expressed systemically, is particularly abundant in skeletal muscle, the heart, adipose tissue, the kidneys, and the lungs (176). FST overexpression through gene transfer or a transgene induces skeletal muscle hypertrophy, myofiber regeneration, and satellite cell proliferation (177, 178). Circulating MSTN and FST are negatively correlated with muscle function in older women (179). In patients with severe muscle atrophy, the MSTN pathway was found to be significantly downregulated with a progressive increase in FST, which may partially delay muscle atrophy (180). Although FST overexpression increases muscle mass and excitability, it does not prevent the age-related decline in motor unit function (177).

Decorin is an exercise-induced muscle factor expressed in various tissues, including intestinal tissue, heart, adipose tissue, and skeletal muscle. It plays a role in regulating autophagy, inflammation, and glucose homeostasis, and has been shown to effectively prevent muscle atrophy by inhibiting MSTN (181). Decorin is an anti-fibrotic and pro-myogenic generating agent. When it is injected into damaged skeletal muscle directly, it can promote the process of complete skeletal muscle regeneration and reduce the formation of fibrotic scar tissue (182). Decorin reduces MSTN-induced phosphorylation of Smad2 and inhibits the activation of the Smad signaling pathway in a dose-dependent manner (183). Intramuscular injection of recombinant Decorin may significantly enhance muscle mass in dystrophic mice by activating skeletal muscle cell differentiation (184, 185). Leukemia inhibitory factor (LIF), which belongs to the IL-16 family, regulates skeletal muscle growth and regeneration and is associated with skeletal muscle after prolonged exercise (49, 186). Aerobic exercise upregulates LIF expression in human skeletal muscle to inhibit myasthenia gravis and improve muscle performance. Exogenous LIF intervention may enable human myoblast proliferation by inducing the cell proliferation factors c-Myc and JunB (49). IL-15 is a contraction-induced myokine that improves energy metabolism in skeletal muscle locally (187, 188). High IL-15 levels protect against high-fat diet-induced obesity, glucose intolerance, and insulin resistance (188). A comparison of wild-type, IL-15 knockout, and IL-15 transgenic mice reveals that IL-15 promotes muscle protein synthesis and myofiber regeneration by activating critical regulators of skeletal muscle autophagy (189, 190). IL-15 and its cognate receptor α (IL-15 receptor α) are involved in the regulation of anabolic and catabolic homeostasis in skeletal muscle. IL-15Rα may play a role in the increased synthesis of myofibrillar proteins in skeletal muscle after a single bout of resistance exercise (191). However, few studies have reported an association between the above muscle factors and sarcopenia in aging sarcopenia, but they all have the function of maintaining muscle mass and enhancing muscle strength.

## 7 Strategies to improve aging sarcopenia

### 7.1 Exercise improves aging sarcopenia

Diminished function and impaired remodeling of motor units are commonly observed in elderly patients with sarcopenia (192). Liu (193) synthesized different exercise intensities in elderly patients with sarcopenia and found that all exercises improved muscle strength and mass, and high intensity was more effective in increasing strength than low or moderate intensity. The high-intensity interval training (HIIT) model, which alternates high-intensity intervals with low-intensity recovery periods, provides physiological benefits quickly (194). HIIT induces transcriptional co-activation of PGC-1α via mTOR and rps6 phosphorylation, which promotes muscle hypertrophy to mitigate skeletal muscle atrophy and enhance overall exercise performance (193). HIIT also enhances locomotor performance in aged mice, which includes muscle mass and strength, grip strength, and mitochondrial biomass (195).

Resistance exercise, also known as weight training and strength training, requires the muscles to resist applied external force or weight. Resistance exercise can improve muscle strength, mass, and physical performance in middle-aged and older adults (196). Twice-weekly resistance exercise is an appropriate prescription for patients with aging sarcopenia (196). A 10-week resistance training in 70-year-old male and female patients with sarcopenia found that the resistance training intervention resulted in an increase in both the mass of lean and limb skeletal muscle (197). The mechanism by which resistance exercise effectively promotes skeletal muscle protein synthesis may involve regulating the secretion of myokines and participating in microRNA regulatory processes (198). Active resistance training also played a favorable role on the lumbar spine, lean body mass, and muscle strength (199). Additionally, resistance training affects visceral fat loss, blood pressure, glucose, and fat metabolism beneficially (200). While numerous studies have documented the beneficial effects of resistance exercise in older individuals with sarcopenia, high-intensity resistance training may not be appropriate for elderly populations and those with lower fitness levels (201). It is important to find ways to improve skeletal muscle mass and strength more safely and effectively while significantly reducing mechanical stress. Low-load resistance training with blood flow restriction (L-BFR) induces similar increases in muscle mass but has less effect on skeletal muscle strength compared to H-RT (high-load resistance training), which is considered an effective countermeasure for sarcopenia (201). Static stretching has the potential to increase skeletal muscle strength and endurance, which leads to significant increases in skeletal muscle suppleness (202).

Now, it is widely acknowledged that physical activity plays a crucial role in the prevention and treatment of sarcopenia (200). Various exercise modalities—including resistance training, aerobic training, and balance training—are all effective in promoting muscle health among older adults. Among them, high-volume, high-intensity resistance training is the most significant in improving skeletal muscle mass and physical function in older adults (200). For elderly patients with sarcopenia, the choice of specific exercise modes should be based on individual characteristics, limitations, and needs, where safety is always a key factor (196, 199).

### 7.2 Nutrients improve aging sarcopenia

A healthy diet with adequate protein, vitamins, antioxidant nutrients, and long-chain unsaturated fatty acids may alleviate sarcopenia (203). Vitamin D/vitamin D receptor signal affects all stages of myogenesis by stimulating skeletal muscle fiber proliferation, differentiation, and maintenance and improving skeletal muscle mass and strength (204, 205). On the one hand, vitamin D directly inhibits skeletal muscle atrophy. On the other hand, it inhibits MSTN expression via IGF-independent signal indirectly, which prevents skeletal muscle degeneration and improves myofilament and muscle strength (204, 205). Chronic hypovitaminosis D may lead to upregulation of muscle atrophy markers (Murf1 and MaFbx), VD receptor loss, and a dramatic reduction in cellular remodeling capacity (205). VD receptor deficiency in mouse myocytes is associated with lean body mass, sarcopenia, reduced grip strength, and exercise capacity (206). Human studies also reported that vitamin D deficiency reduces skeletal muscle grip strength and stride speed, particularly pronounced in the elderly (207, 208), whereas oral nutritional supplementation with protein and vitamin D in elderly patients with sarcopenia effectively enhances skeletal muscle mass, although it does not improve physical mobility (209). However, in older adults with vitamin D deficiency, a longer duration of supplementation or a higher dosage of vitamin D may be necessary compared to younger adults to achieve the beneficial effects on skeletal muscle (204). Vitamin D supplementation during resistance training will improve skeletal muscle mass and positively impact skeletal muscle remodeling in both older and younger adults (210).

Essential amino acids (EAAs) stimulate skeletal muscle protein synthesis and turnover, play a key role in replacing degraded or damaged skeletal muscle proteins, and lay the metabolic foundation for enhanced skeletal muscle function (211). Several studies of EAA supplementation in elderly patients with sarcopenia have found that EAA interventions have a positive effect on both skeletal muscle mass and strength (212). Supplementation of an animal protein diet containing EAA during resistance training synergistically promotes increased skeletal muscle mass (213). Cuyul-Vásquez and coworkers (214) demonstrated that whey protein supplementation during resistance training significantly increased skeletal muscle mass and grip strength in elderly patients with sarcopenia. However, if protein supplementation is stopped at the end of 12 weeks of exercise, it may result in a skeletal muscle protein synthesis decline (215). Appropriate intake of energy, protein, long-chain saturated fatty acids, amino acids, vitamin D, and antioxidants may reduce the decline in skeletal muscle mass and strength in older adults (216). Furthermore, there are also some natural products that have been suggested for the treatment and prevention of sarcopenia, such as ursolic acid and pentacyclic triterpene acid fruits (apple peel and tomatidine) (217). In animal models, these natural products increase muscle mass and grip strength in mice by decreasing age-related muscle atrophy mediator activity (217). β-Hydroxy β-methylbutyrate (HMB) has been shown to improve muscle mass without affecting muscle strength and function in sarcopenic or debilitated older adults (218). β-Sitosterol is widely found in various parts of plants and has various effects, such as anti-inflammatory, anti-alcoholic fatty liver, and antioxidant. It can protect mice from the muscle atrophy induced by dexamethasone (9).

Daily dietary structure may influence protein intake and interfere with skeletal muscle protein metabolism and transcription, thereby accelerating skeletal muscle mass loss in older adults (219). Plant-based dietary patterns are becoming more popular in improving skeletal muscle atrophy, and related studies have shown that plant-based dietary patterns are more beneficial and effective than animal-based dietary patterns in maintaining muscle mass in functionally independent Chinese older adults (220). There is less research on the effects of vegan diets on physical performance and body composition. However, one study of a vegan diet in young women showed that dietary changes would lead to alterations in overall macronutrient compartmentalization that would impair skeletal muscle mass (221). In the elderly population, vegan diets increase the risk of inadequate protein intake, negatively affecting skeletal muscle mass (222).

Aging sarcopenia has attracted widespread attention as a skeletal muscle disease in the elderly, but there are no suitable clinical interventions. Nutrition and exercise are the main methods for its prevention and treatment. Appropriate intake of animal and vegetable proteins and a balanced dietary structure combined with appropriate physical activity are important strategies to improve sarcopenia in the elderly.

## 8 Conclusion

In this paper, we summarize the current state of research on aging sarcopenia and the effects of various exerkines on skeletal muscle. Exercise-induced exerkines improve aging sarcopenia mainly by increasing skeletal muscle energy supply, activating satellite cells involved in skeletal muscle repair and regeneration, improving the function of the neuromuscular junction, and enhancing the neural control of skeletal muscle. Daily dietary changes affect skeletal muscle regeneration and repair by altering protein intake. Therefore, a rational diet combined with exercise training is one of the most effective measures to improve aging sarcopenia. However, there is a lack of research on the mechanisms of combining myokines with nutrients to improve aging sarcopenia. Meanwhile, further research is needed to study the effects of multiple exerkines to improve aging sarcopenia.
